# Developing a Family Medicine Program in Jordan: appraisal of trainees and trainers and final assessment outcomes

**DOI:** 10.12688/mep.20687.1

**Published:** 2024-11-12

**Authors:** Birgitte Schoenmakers, Safiya Virji, Benjamin Colton, Lana Alhalaseh, Jiskoot van Ewijk, Marleen, David Spitaels, Amjad Al Shdaifat

**Affiliations:** 1 Public Health and Primary Care, KU Leuven, Leuven, 3000, Belgium; 2 Queen Mary University of London, London, England, UK; 3 The Hashemite University Faculty of Medicine, Az-Zarqa, Zarqa Governorate, Jordan; 4 The University of Jordan School of Medicine, Amman, Amman Governorate, Jordan; 5 Radboud University, Nijmegen, Gelderland, The Netherlands

**Keywords:** Family Medicine Education, Curriculum Development, Transcultural Collaboration

## Abstract

**Introduction:**

Curriculum development in medical education, particularly in family medicine, is essential for preparing healthcare professionals to meet evolving patient needs. This article examines the development of a Family Medicine program in Jordan, focusing on challenges, methodologies, and outcomes.

**Methods:**

The curriculum was developed through collaboration between Jordanian and European universities, emphasizing core family medicine principles, evidence-based practice, and local context. Two cohorts of trainees participated in the one-year program, which used a 'whole task learning model' covering communication, clinical knowledge, and community health. Feedback was gathered through focus group interviews with trainers and trainees, and pre- and post-test data were analyzed to assess effectiveness.

**Results:**

Feedback indicated positive perceptions among trainers and trainees. Trainees valued the transition to Arabic-led lectures, improved primary care understanding, and trainer engagement. Strengths included evidence-based guidelines and patient interaction emphasis. Areas for improvement included more face-to-face training and practical opportunities. Trainers suggested enhancing practical skills training and increasing Arabic materials. Despite pretest score variations, both cohorts showed significant improvement. Challenges such as non-participation and cheating highlighted the need for regular attendance and academic integrity.

**Conclusion:**

The research underscores the importance of feedback from trainees and trainers in curriculum development. Continuous improvement, comprehensive assessment, and prioritizing linguistic and cultural relevance are crucial for enhancing primary care delivery in Jordan.

## Introduction

Curriculum development in postgraduate medical education is a crucial process that shapes the learning experiences of healthcare professionals ^1^. Effective curriculum development ensures medical education aligns with the evolving needs of healthcare and equips professionals with the necessary skills and knowledge ^2^. Family medicine encompasses a wide range of ages and types of care ^3^. Therefore, the curricula must address the diverse needs of patients across the lifespan. Well-designed curricula ensure that family practitioners receive comprehensive training, adapt to changing healthcare landscapes, and provide high-quality care to their patients ^4^. The curriculum must strike a balance between covering essential topics comprehensively and avoiding overload. Prioritizing content while ensuring depth of understanding is a challenge. Furthermore, providing adequate clinical exposure is crucial. Balancing didactic learning with hands-on experience can be challenging, especially in family medicine where clinical skills are paramount. To make the training successful, equipping family medicine supervisors with the necessary skills to teach family medicine effectively is critical and requires resources and commitment ^1^. Limited time, budget, and trainer availability can hinder curriculum development and training which makes prioritizing the most important topics and training essential.

Curriculum development across cultures presents unique challenges that educators must navigate to create effective and inclusive educational programs ^5^. Adapting curricula to diverse cultural contexts requires understanding the values, norms, and beliefs of different communities. Curricula often carry implicit assumptions about learners’ prior experiences, which may not be held universally, and diverse cultures prioritize various educational philosophies ^6^. Also, health-related beliefs and practices differ significantly worldwide which challenges the development of learning materials ^7^. Finally, there is a risk of imposing a standardized curriculum globally.

Jordanian medical schools currently graduate close to 2000 students annually. This number far exceeds the approximately 400 available residency training positions of which only 30 are for family medicine training. On average, medical students have only two weeks of training in an outpatient primary healthcare setting during their entire medical school education. Due to the lack of residency positions, most graduates complete a mainly unsupervised one-year internship which is inpatient-focused and then directly begin working as General Practitioners (GPs) in an outpatient setting. A substantial portion of these GPs find jobs within the ministry of health (MOH) and often work in rural areas without nearby mentors available.

Jordan's health statistics have improved over the last 50 years, but there remains a great discrepancy in access to care nationwide. While more residency programs are needed in Jordan to train primary care practitioners in both inpatient and outpatient medicine, the reality is that most of the graduates end up practicing in an outpatient setting. We developed a one-year outpatient-based training program to provide GP trainees with enough training to confidently and accurately develop a broad differential diagnosis, decide on a specific diagnosis, and properly treat disease. Emphasis was placed on critical thinking, evidence-based medicine, professionalism, health leadership, and communication skills. The goal of the training program was to increase patient care quality, dealing with the increasing complexity of ware and leading to better health in the entire community.

In this research we describe the results of the development of a postgraduate family medicine program in Jordan. We investigated the experiences of trainers and trainees and analyzed the results of the final exam for two consecutive cohorts of trainees.

## Methods

### Design of the program

The one-year training program targeted general practitioners working in Jordanian primary care Health Centers who have no benefits from any Family Medicine specialization. Trainees went through a formal application procedure, chaired by selection committee members from the consortium, before being admitted to the program. There were two cohorts of trainees (starting in 2021 and 2022). From the Jordanian universities, four faculties were involved. The trainers were all experienced trainers in medical education and clinicians in outpatient settings.

The development of the family medicine program was set up in a consortium of three Jordan universities and three European universities (UK, The Netherlands and Belgium). To build and evaluate this program, we followed the six steps model as described by Patricia Thomas and David Kern ^8^. In a first step, the Jordan universities performed a needs assessment among trainers and GPs. The emphasis of the program needed to be on core principles of family medicine, management of common presentations, professionalism, and an evidence-based approach. Second, we determined and prioritized the content of the curriculum based on the needs of the learners and the goals of the curriculum. We started from eight entrustable professional activities (EPA’s): short episode care, chronic care – multimorbidity, end of life care, mother-child care, emergency care, psychosocial care- mental health, elderly care, and gender related care. Thirdly, we wrote down the learning goals and objectives. The five dimensions of family medicine are the red thread through the EPAs: communication, organisation, clinical knowledge and skills, attitudes and ethics and community and public health/wellbeing. Fourth, appropriate teaching strategies were selected. The ‘whole task learning model’ is an evidence-based teaching and learning model that departs from real life authentic tasks (‘clinical cases’). Each module lasts 4 weeks and includes a preparatory assignment, a lecture, an interactive session with an expert and an integrated workshop. The trainees received background material, interactive material and exercises, continuous self-evaluation and feedback from trainers and peer tutors. The learning process is supported by a digital learning platform (Microsoft Teams). A tailored user manual guided the trainees through the structure, content, and objectives of the learning environment. We divided the different modules between the partners in a close collaboration between Jordan and European partners to search for the best way to educate and coach the Jordan trainees and trainers in the program. Jordan trainers also participated in a train the trainer course. Fifth, the curriculum was rolled out and a group of selected primary health care general practitioners was included in the program. These GP trainees were supervised by trainers on a one-to-one basis through clinic on site observations. In the first year (cohort 1, 2021), all courses were given by the European trainers and co-piloted by the Jordan trainers. In the second year (cohort 2, 2022), the Jordan trainers operated independently from the European trainers. All lectures and seminars took place online in the Teams learning environment. Sixth, we performed an evaluation and determined the effectiveness of the program.

Beside the theoretical training, trainees were also supervised in their clinical work setting by dedicated workplace trainers

### Design of the research

To assess the program's outcome, we defined two research questions: what is the lived experience of trainers and trainees involved in it and what is its impact on the final assessment?

In answer to the first question, we conducted focus group interviews with trainers and trainees after each cohort (2022 and 2023). Interviews were built in accordance with the Coreq checklist for interviews and analyzed following the Quagol guidelines for qualitative data analysis ^9, 10^. The aim of the trainees’ focus groups was to get a feel for the experiences of the trainees involved in the project. For that purpose, we asked questions per aspect of education: What was good about the modules and what could be done better? These questions addressed particularly organization, content, curriculum delivery and learning environment.

For the same purpose, we organized focus groups for the trainers. They were asked to reflect on their role as a teacher, experiences with the program (same themes as trainees) and with the peer sessions, more specifically since this concept was very new and challenging.

The focus group interviews were audiotaped and transcribed. Trainees and trainers participated on a voluntary basis. The focus groups were led by the European partners and supported by the Jordanian faculty members. Arabic interpreting was provided since English proficiency was not always sufficient to delve deeper into themes.

At the beginning and the end of each cohort, trainees took an exam. This exam consisted of 50 multiple choice questions addressing primary care cases. All EPA-themes were equally represented. The exam was composed by both Jordanian and European partners and adjusted to the context. Formative, self-assessment was provided throughout the program and offered via assignments posted in the Teams learning environment. Completion of these exercises was obligatory and followed up by the faculty.

To ensure the research adhered to high-quality standards, we followed the COREQ (Consolidated Criteria for Reporting Qualitative Research) checklist.

## Results

For each cohort we involved five trainers and five trainers in the focus group. The groups were led by a European partner and supported by an Arabic speaking faculty member. Arabic was the main language during the discussions.

In the first cohort and second cohort respectively 25 and 50 candidates applied. Respectively 16 and 27 candidates were admitted by the selection committee. Candidates were all practicing ‘general practitioners in an outpatient clinic’ and aged between 35 and 50 years. The male-female rate was 12:1. Two candidates from the first cohort and one candidate from the second cohort were disenrolled from the program because of not attending sessions.

### Focus groups trainees

First, the idea and concept of the program were welcomed. Trainees mentioned that they learned more than expected and that they were not aware of the very particular nature of primary care. Trainees were also very enthusiastic about the trainers. They praised their expertise, the direct link to clinical practice and the case-based approach. Contact and insights in a care context outside Jordan was also found to be an added value. At the same time, trainees appreciated the adaptation of the learning materials to the local context. Trainees valued the fact that the lecture language was English but noticed that their English proficiency appeared insufficient to properly follow the class. In the second cohort, lectures were led by Arabic speaking Jordan faculty, which was rated as far more convenient. Trainees appreciated the introduction and application of evidence-based primary care guidelines which helped them reduce the number of prescriptions, technical investigations, and unnecessary hospital referrals. Insights into health care systems and population management were also welcomed. Trainees reported that they felt enhanced in their professional competence in general and in primary care in particular. They learned that a patient-doctor's relationship is one of trust and not of a merely instrumental nature. The communication modules and the emphasis on communication skills throughout the course were appreciated by the trainees. They learned to keep an open conversation with patients and to actively listen and experienced the added value of communication models.

Trainees also formulated areas for improvement. They wished that the duration of the face-to-face trainings in contact with the trainers was longer and that the program was extended with one year for practical training. At the same time, they struggled with the concrete planning of the course as they were all scheduled in a busy outpatient clinic. Here, trainees also mentioned that their overcrowded workplace impedes the typically holistic approach. Regarding the content, some trainees lacked the adaptation of the course to the Jordanian context and wished that Arabic were the language used. Topics like mother and childcare and sexual health were less useful since exposure to these patient categories is rare. Here, they suggested adding more psychosocial topics and strategies to talk about health promotion, preconception care and dental hygiene. End of life care was considered as typically secondary care. Guidelines were not always applicable in the Jordanian context, due to prescribing regulations. Finally, trainees regretted that there was little uniformity in the provision of preparatory assignments, which negatively impacted participation.

They also asked for more practice moments and vocational training under supervision. They felt less confident in specific communication themes such as ‘breaking bad news’ and ‘sexual health’.

Some trainees suggested opening more dialogue during the lectures. Also, they mentioned that they lacked feedback on assignments and exercises.

### Focus groups trainers

The trainers rated the learning contents and teaching materials as very acceptable. The topics were believed to be very comprehensive and well presented to the trainees. They believed that the learning content was adapted to the local learning needs. They praised the level of interaction with the trainees and the sharing of common primary care cases. As for the trainees, the introduction of evidence-based medicine and the guidelines was well appreciated.

Trainers suggested introducing more materials and classes in Arabic. They also expressed the need for more in-depth communication and vocational training and to add practical skills training. Here, physical examination and emergency training were mentioned as topics to be added. Some trainers believed that the theoretical content was not basic enough as an introduction to primary care. They also suggested organizing the course in remote cities and not only in the capital and increasing the clinic visits for real-time feedback. Trainers noticed that trainees very often renounced assignments and struggled with on-time participation in the (online) classes. They believed that more pressure and emphasis on the preparatory assignment would help increase participation.

### Assessment

During the course, two trainees in cohort 1 and one in cohort 2 did not attend classes and were expelled from the program.

The average test score on the pretest in cohort 1 was 32/50 (n=10, median 33) and in cohort 2 28.6/50 (n=20; median 36). Four trainees of cohort 1 and five in cohort 2 did not participate in the pretest. In cohort 2, six trainees cheated during the exam and their scores were removed from the analysis.

On the posttest in cohort 1, the average score was 36,4 (n=14; median 30) and in cohort 2 33,4 (n= 26; median 42). Here, all trainees participated but three were excluded from analysis because of cheating.

The difference between pre- and posttests was statistically significant in both cohorts (respective t-test p value 0.06 and 0.001).

After deliberation by the Jordanian trainers respectively 14 and 16 trainees in cohort 1 and 2 graduated and received the Family Medicine Diploma.

## Discussion

From the focus groups, we learned that trainees expressed a positive reception towards the program, highlighting their appreciation for the curriculum's concept and structure. They noted significant learning outcomes beyond their initial expectations, particularly in understanding the unique nature of primary care. The enthusiasm towards trainers underscores the program's success in engaging and leveraging their expertise effectively. The emphasis on case-based learning and exposure to diverse healthcare contexts resonated well with trainees, enhancing their professional competence and communication skills. Indeed, a well-structured curriculum that considers engagement, learning outcomes, and context-specific elements is essential for effective medical education ^11^. Above, collaborative teaching and mentorship play a crucial role for an optimal learning experience ^2^.

The transition from English to Arabic lectures reflects an important adaptation based on trainees' feedback, addressing concerns regarding language proficiency and comprehension. This adjustment demonstrates the program's responsiveness to learners' needs and underscores the importance of linguistic accessibility in educational settings ^5^. Moreover, the appreciation for localized learning materials emphasizes the significance of cultural relevance in curriculum design, fostering greater engagement and applicability among participants ^12^. The adjustment highlights the importance of linguistic accessibility in educational environments. Ensuring that content is comprehensible and culturally relevant enhances learning outcomes ^5^.

Trainees valued the integration of evidence-based primary care guidelines, which facilitated a more judicious approach to medical decision-making and reduced unnecessary interventions. Integrating evidence-based guidelines, understanding global contexts, and emphasizing trustful patient interactions contribute to effective primary care ^4, 11, 13^. Evidence-based guidelines help minimize unnecessary interventions, preventing overtreatment and unnecessary healthcare costs.

The emphasis on the patient-doctor relationship as one of trust reflects a pivotal aspect of primary care, emphasizing empathy and effective communication in medical practice. The emphasis on a trust-based patient-doctor relationship is pivotal in primary care. Empathy and effective communication foster better patient outcomes ^4, 14^.

Trainees identified several areas for improvement, including the need for extended face-to-face training duration and practical training opportunities. In the daily routines of family physicians, the electronic patient record has evolved beyond a mere documentation tool. It now actively assists family physicians in delivering well-structured medical interventions. When designing a curriculum, one might consider examining the electronic medical record systems employed locally. By incorporating commonly used systems into teaching modules, we can enhance the transition from theoretical knowledge to practical application ^14, 15^. Challenges related to scheduling within busy outpatient clinics highlight the importance of optimizing learning environments to support holistic education ^12^. Suggestions for curriculum enhancement, particularly in psychosocial topics and practical skills training, underscore the evolving needs of primary care practitioners in addressing diverse patient needs effectively ^4^. Another challenge we encountered was the lack of consistency in the preparatory assignments. In a multifaceted learning environment, it is crucial to have well-defined tasks that promote active participation and enhance the learning process ^13^.

Trainers acknowledged the comprehensive nature of the curriculum and its alignment with local learning needs. However, they identified opportunities for improvement, such as the inclusion of more Arabic materials and enhanced emphasis on practical skills training. This might indeed positively impact accessibility to the program ^11^. Suggestions for increasing participation and engagement through preparatory assignments reflect a proactive approach to optimizing learning outcomes. Emphasizing preparatory assignments may improve engagement ^16^.

Finally, three trainees did not attend classes and were subsequently expelled. This highlights the importance of regular attendance and engagement in the educational process. Rewarding participation might stimulate students to adhere to the learning process and to increase effective involvement in the program ^11^.

The variation in pretest scores suggests that curriculum developers should assess the adequacy of pre-course preparation and consider targeted interventions for trainees with lower baseline knowledge ^1^. Above, curriculum planners should explore reasons for non-participation and implement strategies to ensure comprehensive assessment coverage ^12^. Several trainees cheated during the exam by using resources. In the future, there must be emphasis on academic integrity and on mechanisms to prevent cheating. Both cohorts demonstrated improvement, indicating the effectiveness of the curriculum. However, the variation in median scores warrants further investigation. The difference between pre- and posttests was statistically significant in both cohorts. Here, the curriculum designers should explore the specific areas of improvement and identify successful teaching methods ^2^.

## Conclusion

In conclusion, these results offer valuable insights into the strengths and areas for improvement of the primary care training program in Jordan. By incorporating feedback from both trainees and trainers, the program can evolve to better meet the evolving needs of primary care practitioners, fostering a culture of continuous learning and professional development in the healthcare landscape. There is a need for a well-structured curriculum addressing attendance, assessment integrity, and continuous improvement to enhance educational outcomes. Also, strategies must be implemented to ensure comprehensive assessment coverage.

Future iterations of the program should prioritize linguistic accessibility, cultural relevance, and practical skill development to ensure its continued success in enhancing primary care delivery in Jordan.

## Ethics and consent

The program was developed in the context of a Capacity Building in Higher Education (202). All data were collected as part of the program's evaluation and served as quality indicators for reporting to the contractor. Focus groups were organized to collect data for the interim and final reports. These data and analyses were used for reporting in this manuscript. Trainers and trainees voluntarily took part in the focus group and were informed about the data collection's purpose and verbally consented to participate and share data for reporting and research purposes. Consent for participation was obtained at the beginning of the project and was associated with the formal registration in the program. All data were processed and analyzed anonymously. The data were gathered during the program’s evaluation, commissioned by the funding organization, and utilized in both interim and final reports. Subsequent analysis of the data was conducted retrospectively, thus ethical approval was not required.

## Data Availability

***Underlying data*** KU Leuven RDR: Developing a family medicine program in Jordan: appraisal of trainees and trainers and final assessment outcomes https://doi.org/10.48804/FZCNFZ ^17^ The project contains the following underlying data: 1. Pre and post test scores for both cohorts anon.xlsx 2. README_template Jordan Fam Med Prog.txt 3. Transcription focusgroup trainees.docx Data are available under the terms of the Creative Commons Attribution 4.0 International license (CC-BY 4.0).
