# Supplementary material for: Developing a Family Medicine Program in Jordan: appraisal of trainees and trainers and final assessment outcomes
Source: MedEdPublish (2016). 2025 Aug 22;14:276. Originally published 2024 Nov 12. [Version 4] doi: 10.12688/mep.20687.4 (PMC12166353; doi:10.12688/mep.20687.4)
Supplement: Peer review history

# Reviewer report: Reviewer response for version 4

**DOI:** 10.21956/mep.22636.r43177

**Published:** 2025-08-30

**Authors:** Jacqueline Torti

**Affiliations:** 1 Western University, London, Ontario, Canada

No further comments.

Is the case presented with sufficient detail to be useful for teaching or other practitioners?

Partly

Is the work clearly and accurately presented and does it cite the current literature?

Partly

If applicable, is the statistical analysis and its interpretation appropriate?

Partly

Is the case’s background and context in sufficient detail?

Partly

Are all the source data underlying the results available to ensure full reproducibility?

Yes

Are the conclusions drawn adequately supported by the results?

Yes

Reviewer Expertise:

Expertise in family medicine, medical education, curriculum development and qualitative inquiry.

I confirm that I have read this submission and believe that I have an appropriate level of expertise to confirm that it is of an acceptable scientific standard.

# Reviewer report: Reviewer response for version 3

**DOI:** 10.21956/mep.22539.r41674

**Published:** 2025-06-13

**Authors:** Jacqueline Torti

**Affiliations:** 1 Western University, London, Ontario, Canada

Thank you for the opportunity to review the revised manuscript. I thank the authors for their revisions. These changes have improved the manuscript’s clarity and structure. Upon further review, however, there are still a few areas where additional detail or clarification would enhance the transparency and methodological rigor of the work. Please find my follow-up comments below.

**Methods:**

- Previous Comment: In the first paragraph of the Methods section (under Design of the Program), you reference the “whole task learning model” as an evidence-based teaching approach. It would be helpful to include a citation or two to support this educational framework for readers unfamiliar with the model.
- Author Response: True and we added it now also in the method section.
- Follow-Up: I don’t see a citation added here and you don’t mention the “whole task learning model” in the Methods section. Could you appropriately cite the following sentence that uses the definition of this learning model: “The ‘whole task learning model’ is an evidence-based teaching and learning model that departs from real life authentic tasks (‘clinical cases’).”

 

- Previous Comment: The end of the same paragraph refers to “Supplementary Materials 1.” If this is one of the links listed in the Availability of Data section, could you clarify and label it accordingly? It’s currently unclear which material is being referenced.
- Author Response: I searched the manuscript but did not find this ‘supplementary materials 1’ in my version.
- Follow-Up: The last sentence of the first paragraph of the Methods section states, “Classes and workshops took place on a weekly base and lasted six hours (suppl materials 1).” If this is one of the links listed in the Availability of Data section, could you clarify and label it accordingly? It’s currently unclear which material is being referenced.

 

- Previous Comment: The manuscript mentions peer sessions—is this the same as the peer-tutor model mentioned earlier? Could you expand on what these sessions involved and their role in the program?
- Author Response: Correct remark, adjust to peers and not ‘tutos’.
- Follow-Up:  A brief description of what these peer sessions entailed would be helpful. What did the peer sessions involve?

**Results:**

- Previous Comment: How were the individual participants for the focus groups recruited and selected? Some additional detail on sampling or recruitment strategy would strengthen the trustworthiness of this section.
- Author Response: Added: personal approach.
- Follow-Up: Could you provide additional details on how participants were selected?

Overall, the manuscript presents an interesting and important contribution. The data are compelling and the revisions have clarified several aspects of the study. Addressing the points above—particularly with respect to consistency in terminology, citation of frameworks, and more detailed methodological description—will further improve the transparency and clarity of the manuscript.

Is the case presented with sufficient detail to be useful for teaching or other practitioners?

Partly

Is the work clearly and accurately presented and does it cite the current literature?

Partly

If applicable, is the statistical analysis and its interpretation appropriate?

Partly

Is the case’s background and context in sufficient detail?

Partly

Are all the source data underlying the results available to ensure full reproducibility?

Yes

Are the conclusions drawn adequately supported by the results?

Yes

Reviewer Expertise:

Expertise in family medicine, medical education, curriculum development and qualitative inquiry.

I confirm that I have read this submission and believe that I have an appropriate level of expertise to confirm that it is of an acceptable scientific standard.

# Reviewer report: Reviewer response for version 2

**DOI:** 10.21956/mep.22438.r41106

**Published:** 2025-04-14

**Authors:** Jacqueline Torti

**Affiliations:** 1 Western University, London, Ontario, Canada

Thank you for the opportunity to review this revised manuscript. I appreciate the thoughtful effort the authors have taken to address the initial feedback, revise the manuscript, and provide a clear and respectful response to reviewer comments. Upon further review, there remain a few areas that would benefit from additional clarification or elaboration to strengthen the manuscript further.

**Abstract:**

- In the results section of the abstract, the sentence: “Despite pretest score variations, both cohorts showed significant improvement,” would be clearer if you specify that the improvement was determined based on post-test examination results.

**Introduction:**

- Thank you for adding additional citations to support parts of the introduction.
- There are still some contextual descriptions related to the Jordanian medical education system—particularly in paragraphs 3 and 4—that would benefit from citations. For instance, statistics about the number of graduates, available residency positions, nature of outpatient training, internship structures, GP practice locations, and broader trends in health statistics are important background details, but its unclear what sources are being referenced. Including appropriate citations here would enhance the credibility of this section.

**Methods:**

- Previous Comment: The learning goals and objectives of the curriculum are mentioned but not detailed. Including them would help readers assess whether the curriculum met its intended outcomes. *We fully understand this comment but the primary focus of this research report was the appraisal of the program by trainers and trainees. We considered that describing ad assessing the learning goals and objectives would lead us to far from the primary research aim. *
  - Response: I appreciate your perspective that including this information might deviate from the paper’s primary focus—namely, the appraisal by trainees and trainers. That said, learning goals and objectives are often foundational to participants’ experiences and perceptions of a program. If you believe including them would distract from your core aims, I respect that decision; however, a brief mention or summary might still help contextualize the appraisals for the reader.

- In the first paragraph of the Methods section (under *Design of the Program*), you reference the “whole task learning model” as an evidence-based teaching approach. It would be helpful to include a citation or two to support this educational framework for readers unfamiliar with the model.
- The end of the same paragraph refers to “Supplementary Materials 1.” If this is one of the links listed in the *Availability of Data* section, could you clarify and label it accordingly? It’s currently unclear which material is being referenced.
- Previous Comment: The third paragraph under ‘Design of the Program’ appears to be incomplete. Could you either remove or integrate this into other paragraphs? *We made the link more clear now. The paragraph insert was confusing.*
  - Response: Thank you for working to improve the coherence of this section. However, the current structure (a single sentence) still feels a bit disconnected from the surrounding content. Would you consider integrating this sentence into the preceding or following paragraph to improve the flow?
- Previous Comment: Program Evaluation: The stated goal is to assess the program’s outcomes, but in another section, you mention assessing program effectiveness. Could you clarify the primary objective of the research? *True and adjusted in both the introduction and the methods section.*
  - Response: You noted this was adjusted, but I was unable to identify the corresponding changes in the Introduction. Would you mind pointing out where the revisions were made? 
- Previous Comment: The manuscript mentions peer sessions—is this the same as the peer-tutor model mentioned earlier? Could you expand on what these sessions involved and their role in the program? *Correct remark, adjust to peers and not ‘tutos’.*
  - Response: Thank you for the update. However, the term “peer-tutor” still appears in the Methods section. A consistent term and brief description of what these sessions involved would be helpful.

**Results:**

- How were the individual participants for the focus groups recruited and selected? Some additional detail on sampling or recruitment strategy would strengthen the trustworthiness of this section.
- Thank you for incorporating quotes from the focus groups—they add valuable depth and richness. One point of clarification: What do the letters “T,” “B,” and “A” preceding the quotes refer to? Providing a legend or brief explanation would help readers follow along. Additionally, attributing quotes more specifically (e.g., "Cohort 1, Focus Group Participant 2") would enhance clarity and transparency.

**Discussion:**

- It would be helpful to explicitly acknowledge any limitations of your study design. For example, were there potential biases due to self-selection of focus group participants, or any challenges with generalizability? Including a limitations section would help contextualize the findings for readers.

Again, I commend the authors for their commitment to strengthening the manuscript. I hope these final suggestions are helpful as you move toward the next stage of revision.

Is the case presented with sufficient detail to be useful for teaching or other practitioners?

Partly

Is the work clearly and accurately presented and does it cite the current literature?

Partly

If applicable, is the statistical analysis and its interpretation appropriate?

Partly

Is the case’s background and context in sufficient detail?

Partly

Are all the source data underlying the results available to ensure full reproducibility?

Yes

Are the conclusions drawn adequately supported by the results?

Yes

Reviewer Expertise:

Expertise in family medicine, medical education, curriculum development and qualitative inquiry.

I confirm that I have read this submission and believe that I have an appropriate level of expertise to confirm that it is of an acceptable scientific standard, however I have significant reservations, as outlined above.

# Response

**Published:** 2025-04-17

**Authors:** Birgitte Schoenmakers

**Affiliations:** Public Health and Primary Care, KU Leuven, Leuven, Flanders, Belgium

Reviewers’ comments 3 : **Developing a Family Medicine Program in Jordan: appraisal of trainees and trainers and final assessment outcomes**

 Dear Reviewer,

 Thank you taking your time again to review this manuscript. I do believe that by now, we addressed all your concerns and remarks.

 Kind Regards,

 Birgitte

**Abstract:**

- In the results section of the abstract, the sentence: “Despite pretest score variations, both cohorts showed significant improvement,” would be clearer if you specify that the improvement was determined based on post-test examination results.

**Introduction:**

- Thank you for adding additional citations to support parts of the introduction.
- There are still some contextual descriptions related to the Jordanian medical education system—particularly in paragraphs 3 and 4—that would benefit from citations. For instance, statistics about the number of graduates, available residency positions, nature of outpatient training, internship structures, GP practice locations, and broader trends in health statistics are important background details, but its unclear what sources are being referenced. Including appropriate citations here would enhance the credibility of this section.

***Author Response: **The Jordan Project partners supplied the data, which is rarely accessible for external use. We supplemented it with publicly available resources.*

**Methods:**

- Previous Comment: The learning goals and objectives of the curriculum are mentioned but not detailed. Including them would help readers assess whether the curriculum met its intended outcomes. *We fully understand this comment but the primary focus of this research report was the appraisal of the program by trainers and trainees. We considered that describing ad assessing the learning goals and objectives would lead us to far from the primary research aim.*

- Response: I appreciate your perspective that including this information might deviate from the paper’s primary focus—namely, the appraisal by trainees and trainers. That said, learning goals and objectives are often foundational to participants’ experiences and perceptions of a program. If you believe including them would distract from your core aims, I respect that decision; however, a brief mention or summary might still help contextualize the appraisals for the reader.

***Author response: **We added more information on how we used the EPAs to formulate the learning goals. We hope this will meet your remark.*

  

- In the first paragraph of the Methods section (under *Design of the Program*), you reference the “whole task learning model” as an evidence-based teaching approach. It would be helpful to include a citation or two to support this educational framework for readers unfamiliar with the model.

**​ *Author response:*** *True and we added it now also in the method section.*

  

- The end of the same paragraph refers to “Supplementary Materials 1.” If this is one of the links listed in the *Availability of Data* section, could you clarify and label it accordingly? It’s currently unclear which material is being referenced.

***Author response: **I searched the manuscript but did not find this ‘supplementary materials 1’ in my version.*

- Previous Comment: The third paragraph under ‘Design of the Program’ appears to be incomplete. Could you either remove or integrate this into other paragraphs? *We made the link more clear now. The paragraph insert was confusing.*
- Response: Thank you for working to improve the coherence of this section. However, the current structure (a single sentence) still feels a bit disconnected from the surrounding content. Would you consider integrating this sentence into the preceding or following paragraph to improve the flow?

***Author response: **Done!*

  

- Previous Comment: Program Evaluation: The stated goal is to assess the program’s outcomes, but in another section, you mention assessing program effectiveness. Could you clarify the primary objective of the research? *True and adjusted in both the introduction and the methods section.*
- Response: You noted this was adjusted, but I was unable to identify the corresponding changes in the Introduction. Would you mind pointing out where the revisions were made? 

***Author response: **Introduction: ‘In this research we describe the results of the development of a postgraduate family medicine program in Jordan. Therefore, we investigated the experiences of trainers and trainees and analyzed the results of the final exam for two consecutive cohorts of trainees. ‘ Methods: ‘we performed an evaluation and determined the effectiveness of the program in terms of exam outcome’*

  

- Previous Comment: The manuscript mentions peer sessions—is this the same as the peer-tutor model mentioned earlier? Could you expand on what these sessions involved and their role in the program? *Correct remark, adjust to peers and not ‘tutos’.*
- Response: Thank you for the update. However, the term “peer-tutor” still appears in the Methods section. A consistent term and brief description of what these sessions involved would be helpful.

***Author response: **Removed indeed.*

**Results:**

- How were the individual participants for the focus groups recruited and selected? Some additional detail on sampling or recruitment strategy would strengthen the trustworthiness of this section.

***Author response: **Added: personal approach.*

  

- Thank you for incorporating quotes from the focus groups—they add valuable depth and richness. One point of clarification: What do the letters “T,” “B,” and “A” preceding the quotes refer to? Providing a legend or brief explanation would help readers follow along. Additionally, attributing quotes more specifically (e.g., "Cohort 1, Focus Group Participant 2") would enhance clarity and transparency.

***Author response: **Initials of their names.*

**Discussion:**

- It would be helpful to explicitly acknowledge any limitations of your study design. For example, were there potential biases due to self-selection of focus group participants, or any challenges with generalizability? Including a limitations section would help contextualize the findings for readers.

***Author response: **True, and done.*

# Reviewer report: Reviewer response for version 1

**DOI:** 10.21956/mep.22147.r40849

**Published:** 2025-02-04

**Authors:** Jacqueline Torti

**Affiliations:** 1 Western University, London, Ontario, Canada

Thank you for the opportunity to review this manuscript. The authors describe the development of a **Family Medicine curriculum in Jordan**, designed through collaboration between Jordanian and European universities. The study evaluates the program’s effectiveness using **focus group interviews and pre- and post-test analyses**. Results indicate **positive trainee and trainer perceptions**, with some areas identified for improvement. The authors emphasize the **importance of continuous curriculum refinement** to enhance **primary care training in Jordan**.

 Overall, this is an **important and relevant study**, but **some areas could be further clarified, expanded upon, or better supported by existing literature**. Below, I provide specific comments and questions, organized by section, that may help strengthen the manuscript.

**Introduction**

- Some claims in the introduction would be **stronger with supporting literature**. For example, the statement about **Jordan’s health statistics improving over the past 50 years** should be cited. Could you include references to substantiate this and other key claims?
- A careful review of the introduction to **identify areas where additional citations are needed** would improve the credibility of the background section.

**Methods**

- **Program Design:** The structure of this section could be **rearranged for better clarity**. The **first paragraph **describes the participants before the program itself—would it be possible to first **outline the program’s structure, duration, and development process**, and then describe the participants?
- You mention following a **six-step model** and conducting a **needs assessment** among trainers and general practitioners. How was this needs assessment conducted? **Could you briefly describe the steps taken**?
- The **learning goals and objectives** of the curriculum are mentioned but not detailed. Including them would **help readers assess whether the curriculum met its intended outcomes**.
- Could you **expand on the program’s duration and format**? Was it **in-person, hybrid, or online**? How many hours per week were trainees engaged?
- The **third paragraph under ‘Design of the Program’** appears to be **incomplete**. Could you either remove or integrate this into other paragraphs?
- **Program Evaluation:** The stated goal is to **assess the program’s outcomes**, but in another section, you mention assessing **program effectiveness**. Could you **clarify the primary objective** of the research?
- In the third paragraph, you state: *“For the same purpose, we organized focus groups for the trainers.”* Could you **clarify what you mean by “the same purpose”**? Are you referring to exploring their experiences, as with the trainee focus groups?
- You mention that trainers were asked to reflect on *“the same themes as the trainees.”* Do you mean they were asked **the same set of guiding questions**? Clarifying this would improve transparency.
- The manuscript mentions **peer sessions**—is this the same as the **peer-tutor model** mentioned earlier? Could you **expand on what these sessions involved** and their role in the program?
- Formative **self-assessment** is mentioned but not revisited later in the manuscript. **Did these assessments contribute to understanding the program’s impact**?
- Regarding the pre- and post-exams:
  - Were the **same test questions used**? If so, could this have influenced score improvements?
  - What **language were the exams written in**? If it was not the trainees’ primary language, could this have influenced their scores?
  - Were there **overlaps between focus group findings and exam results**?
  - How did the **self-assessments contribute** to evaluating the program?

**Results**

- **Focus Groups:** Could you **include direct quotes from transcripts** to support findings?
- **Assessment Section:** You mention that **six trainees were caught cheating**—could you **provide more context on how this was determined**?
- At the end of the results section, you discuss the **number of graduates per cohort**. How was graduation determined? Was it based solely on **exam scores or additional criteria**?

**Discussion**

- How was it determined that trainees **cheated or used external resources** during the exam? Were there **protocols in place to monitor the exam**?
- The manuscript states that **test scores improved, indicating an effective curriculum**. However, could some of this improvement be due to **repetition of the same test**? A discussion of **potential test-retest effects** would be useful.
- Did participant **test scores align with their feedback in focus groups**? Were there **any discrepancies** between their reported experiences and measured outcomes?

**Overall Feedback**

 This manuscript presents an **important study on curriculum development in Family Medicine in Jordan**. The research is **timely and relevant**, but some sections could **benefit from greater clarity, additional detail, and stronger citation support**. The **Methods section, in particular, would be strengthened by a clearer structure and more details about assessment tools and data collection**. Including **quotes from focus groups** and addressing **test-retest effects** would also improve the rigor of the results and discussion.

 Thank you again for the opportunity to review this work. I look forward to seeing how the manuscript develops.

Is the case presented with sufficient detail to be useful for teaching or other practitioners?

Partly

Is the work clearly and accurately presented and does it cite the current literature?

Partly

If applicable, is the statistical analysis and its interpretation appropriate?

Partly

Is the case’s background and context in sufficient detail?

Partly

Are all the source data underlying the results available to ensure full reproducibility?

Yes

Are the conclusions drawn adequately supported by the results?

Yes

Reviewer Expertise:

Expertise in family medicine, medical education, curriculum development and qualitative inquiry.

I confirm that I have read this submission and believe that I have an appropriate level of expertise to confirm that it is of an acceptable scientific standard, however I have significant reservations, as outlined above.

# Response

**Published:** 2025-02-17

**Authors:** Birgitte Schoenmakers

**Affiliations:** Public Health and Primary Care, KU Leuven, Leuven, Flanders, Belgium

Thank you for taking the time to read and review this article. Your comments significantly contributed to improvement of the manuscript.

**Introduction**

- Some claims in the introduction would be **stronger with supporting literature**. For example, the statement about **Jordan’s health statistics improving over the past 50 years** should be cited. Could you include references to substantiate this and other key claims? We added more references now
- A careful review of the introduction to **identify areas where additional citations are needed** would improve the credibility of the background section. Please, see above

**Methods**

- **Program Design:** The structure of this section could be **rearranged for better clarity**. The **first paragraph **describes the participants before the program itself—would it be possible to first **outline the program’s structure, duration, and development process**, and then describe the participants? Adjusted
- You mention following a **six-step model** and conducting a **needs assessment** among trainers and general practitioners. How was this needs assessment conducted? **Could you briefly describe the steps taken**? We added a short note to clarify.
- The **learning goals and objectives** of the curriculum are mentioned but not detailed. Including them would **help readers assess whether the curriculum met its intended outcomes**. We fully understand this comment but the primary focus of this research report was the appraisal of the program by trainers and trainees. We considered that describing ad assessing the learning goals and objectives would lead us to far from the primary research aim.
- Could you **expand on the program’s duration and format**? Was it **in-person, hybrid, or online**? How many hours per week were trainees engaged? The format is mentioned on page 4, from step 4 and on. We added a note to providing more details on duration and engagement.
- The **third paragraph under ‘Design of the Program’** appears to be **incomplete**. Could you either remove or integrate this into other paragraphs? We made the link more clear now. The paragraph insert was confusing.
- **Program Evaluation:** The stated goal is to **assess the program’s outcomes**, but in another section, you mention assessing **program effectiveness**. Could you **clarify the primary objective** of the research? True and adjusted in both the introduction and the methods section
- In the third paragraph, you state: *“For the same purpose, we organized focus groups for the trainers.”* Could you **clarify what you mean by “the same purpose”**? Are you referring to exploring their experiences, as with the trainee focus groups? Indeed, confusing and clarified now.
- You mention that trainers were asked to reflect on *“the same themes as the trainees.”* Do you mean they were asked **the same set of guiding questions**? Clarifying this would improve transparency. Indeed and adjusted
- The manuscript mentions **peer sessions**—is this the same as the **peer-tutor model** mentioned earlier? Could you **expand on what these sessions involved** and their role in the program? Correct remark, adjust to peers and not ‘tutos’
- Formative **self-assessment** is mentioned but not revisited later in the manuscript. **Did these assessments contribute to understanding the program’s impact**? The formative self-assessment consisted of exercises offered as part of the learning materials via the online learning platform. The impact of these exercises was not particularly questioned and trainees did not mention it during the focus groups.
- Regarding the pre- and post-exams:
  - Were the **same test questions used**? If so, could this have influenced score improvements? No, these were different exams
  - What **language were the exams written in**? If it was not the trainees’ primary language, could this have influenced their scores? Arabic
  - Were there **overlaps between focus group findings and exam results**? We did not investigate that since data of both studies were analyzed anonymously.  Trainees did not mention the exams either during the focus groups.
  - How did the **self-assessments contribute** to evaluating the program? The formative self-assessment was not brought up during the focus groups so we can not answer this question.

**Results**

- **Focus Groups:** Could you **include direct quotes from transcripts** to support findings? Done
- **Assessment Section:** You mention that **six trainees were caught cheating**—could you **provide more context on how this was determined**? ‘Several trainees cheated during the exam by using resources during the exam’
- At the end of the results section, you discuss the **number of graduates per cohort**. How was graduation determined? Was it based solely on **exam scores or additional criteria**? Completion of the exercises and attending the classes was obligatory and followed up by the faculty. We added ‘After deliberation, considering the exam results, class attendance and completion of the online exercises,  by the Jordanian trainers respectively 14 and 16 trainees in cohort 1 and 2 graduated and received the Family Medicine Diploma.’

**Discussion**

- How was it determined that trainees **cheated or used external resources** during the exam? Were there **protocols in place to monitor the exam**? This was an exam with live supervision
- The manuscript states that **test scores improved, indicating an effective curriculum**. However, could some of this improvement be due to **repetition of the same test**? A discussion of **potential test-retest effects** would be useful. Agree, but further clarified in answer to a question above: both exams differed substantially.
- Did participant **test scores align with their feedback in focus groups**? Were there **any discrepancies** between their reported experiences and measured outcomes? Agree with this remark, please see also answer to similar remark above

# Reviewer report: Reviewer response for version 1

**DOI:** 10.21956/mep.22147.r40189

**Published:** 2024-12-02

**Authors:** Hiba Salah Abdelgadir

**Affiliations:** 1 Alzaiem Alazhari University, Khartoum, Khartoum, Sudan

The manuscript is well written. It accepted with Minor corrections. 

 I suggest the following changes in the title: **Developing a Family Medicine Program in Jordan: appraisal of trainees and trainers and final assessment outcomes**

1. In Methods section: Please start this section with **Study design and population: **Here you need to talk briefly about the study design, population (selection criteria, total number) and study duration.
2. In Methods section: you need to talk briefly about the study area to let the readers more aware about Jordan.
3. You mentioned in the design of the program section that “The one-year training program targeted general practitioners working in Jordanian primary care Health Centers who have no benefits from any Family Medicine specialization”. What do you mean by *have no benefits*? Do you mean haven’t enrolled in family medicine training? Please clarify.
4. Please specify, Is the trainers were family physicians, or no? And if they weren’t family physicians, why you didn’t involve family medicine trainers?
5. You need to talk briefly about the designed curriculum (Total hours, name of the courses or designed lectures). If it is possible, attach a copy of the designed curriculum as a supplementary material and cite it in the main text.
6. You mentioned that: “In the second cohort, lectures were led by Arabic speaking Jordan *faculty*”. Do you mean Jordan Arabic speaker faculty member/ trainer? Please rewrite in a clear way.

Is the case presented with sufficient detail to be useful for teaching or other practitioners?

Yes

Is the work clearly and accurately presented and does it cite the current literature?

Yes

If applicable, is the statistical analysis and its interpretation appropriate?

Not applicable

Is the case’s background and context in sufficient detail?

Yes

Are all the source data underlying the results available to ensure full reproducibility?

Partly

Are the conclusions drawn adequately supported by the results?

Yes

Reviewer Expertise:

Adherence to the guidelines, guidelines implementation, Curriculum evaluation, psychological impact.

I confirm that I have read this submission and believe that I have an appropriate level of expertise to confirm that it is of an acceptable scientific standard.

# Response

**Published:** 2025-02-17

**Authors:** Birgitte Schoenmakers

**Affiliations:** Public Health and Primary Care, KU Leuven, Leuven, Flanders, Belgium

Thank you for reviewing this article and contributing to an improved version!

1. In Methods section: Please start this section with **Study design and population: **Here you need to talk briefly about the study design, population (selection criteria, total number) and study duration. Agree and adjusted
2. In Methods section: you need to talk briefly about the study area to let the readers more aware about Jordan. Can I refer to the introduction, third paragraph?
3. You mentioned in the design of the program section that “The one-year training program targeted general practitioners working in Jordanian primary care Health Centers who have no benefits from any Family Medicine specialization”. What do you mean by *have no benefits*? Do you mean haven’t enrolled in family medicine training? Please clarify. True, this has been replaced by :’The one-year training program targeted general practitioners working in Jordanian primary care Health Centers **who do not have access** to any Family Medicine specialization’
4. Please specify, Is the trainers were family physicians, or no? And if they weren’t family physicians, why you didn’t involve family medicine trainers? In Jordan, general practitioners and family medicines are not the same. See also introduction:. ‘Due to the lack of residency positions, most graduates complete a mainly unsupervised one-year internship which is inpatient-focused and then directly begin **working as General Practitioners (GPs**) in an outpatient setting. A substantial portion of these GPs find jobs within the ministry of health (MOH) and often work in rural areas without nearby mentors available. **These GPs are not certified as family medicine doctors.  **
5. You need to talk briefly about the designed curriculum (Total hours, name of the courses or designed lectures). If it is possible, attach a copy of the designed curriculum as a supplementary material and cite it in the main text. We added: ‘The full program consisted of 10 themes, one theme a month. Each theme was defined as a EPA. Classes and workshops took place on a weekly base and lasted six hours’ ‘(see suppl materials 1). 
6. You mentioned that: “In the second cohort, lectures were led by Arabic speaking Jordan *faculty*”. Do you mean Jordan Arabic speaker faculty member/ trainer? Please rewrite in a clear way. Indeed, added ‘ **trainers’**
